# Enhancement of Colorimetric pH-Sensitive Film Incorporating *Amomum tsao-ko* Essential Oil as Antibacterial for Mantis Shrimp Spoilage Tracking and Fresh-Keeping

**DOI:** 10.3390/foods13111638

**Published:** 2024-05-24

**Authors:** Yunxia He, Yuan Yuan, Yuanyuan Gao, Mianhong Chen, Yingying Li, Ying Zou, Liangkun Liao, Xiaotong Li, Zhuo Wang, Jihua Li, Wei Zhou

**Affiliations:** 1 Key Laboratory of Tropical Crop Products Processing of Ministry of Agriculture and Rural Affairs, Agricultural Products Processing Research Institute, Chinese Academy of Tropical Agricultural Sciences, Zhanjiang 524001, China; heyunxia63682@163.com (Y.H.); yyuan@catas.cn (Y.Y.); fanny_gao@126.com (Y.G.); mianhong_chen@163.com (M.C.); qinghuan915@foxmail.com (Y.L.); m18998368641@163.com (Y.Z.); liaoliangkunv@163.com (L.L.); lxt20211224@163.com (X.L.); foodpaper@126.com (J.L.); 2 College of Food Science & Technology, Huazhong Agricultural University, Wuhan 430070, China; 3 College of Food Science and Technology, Guangdong Ocean University, Zhanjiang 524088, China

**Keywords:** smart film, *Amomum tsao-ko* essential oil, antimicrobial properties, mantis shrimp preservation, freshness monitoring

## Abstract

Anthocyanin-based smart packaging has been widely used for food freshness monitoring, but it cannot meet the requirements of smart films with antibacterial properties. This study aimed to enhance the antibacterial properties of intelligent films by incorporating *Amomum tsao-ko* essential oil (AEO) for mantis shrimp spoilage tracking and keeping the product fresh. A smart film was designed by introducing AEO and purple potato anthocyanin (PPA) to a polyvinyl alcohol/cellulose nanocrystal (PVA/CNC) polymer matrix. Our findings revealed that APP and AEO imparted the smart film with a favorable oxygen barrier, UV protection, mechanical properties, and antioxidant and pH/NH_3_-sensitive functions. Interestingly, the PVA/CNC-AEO-PPA film achieved 45.41% and 48.25% bactericidal efficacy against *S. putrefaciens* and *V. parahaemolyticus*, respectively. Furthermore, a visual observation confirmed that the target film (PVA/CNC-AEO-PPA) changed color significantly during mantis shrimp spoilage: rose red—light red—pink—light gray—dark gray. Meanwhile, the PVA/CNC-AEO-PPA film retarded the quality deterioration of the mantis shrimp effectively. The PVA/CNC-AEO-PPA film shows great application potential in mantis shrimp preservation and freshness monitoring; it is expected to become a rapid sensor for detecting seafood quality non-destructively and a multifunctional film for better preservation of product quality.

## 1. Introduction

Mantis shrimp (*Oratosquilla oratoria*) is popular among consumers for its high-quality proteins and delicious taste. However, mantis shrimp are susceptible to spoilage caused by enzymatic reactions and microbial contamination, thereby resulting in adverse effects on human health [1]. Standard laboratory-level methods for detecting seafood freshness are destructive, time-consuming, and require expensive and precise instruments [2]. The search for a novel, quick, non-destructive method to detect seafood spoilage is increasing in momentum [3].

When seafood undergoes spoilage, significant amounts of TVB-N are produced through microbial degradation. The accumulation of TVB-N leads to a gradual rise in the pH value of the package [4]. Therefore, intelligent films with pH-sensing ability can reflect the freshness levels of the packaged seafood by generating a reliable color response [5]. These films can be sourced from a variety of materials, such as polysaccharides, lipids, and proteins. Chemical synthetic dyes such as bromocresol green, methyl red, and bromocresol purple have been widely used in the manufacture of intelligent packaging [6]. Nevertheless, the potential toxicity of these chemo-sensitive compounds limits their application in food quality assessment. Hence, non-toxic natural anthocyanins are the preferred pH indicators in food packaging systems for their superior antioxidant and pH-sensitive properties [7]. Compared to other anthocyanins, purple potato anthocyanins (PPA) exhibit greater heat/light stability and a broader pH-mediated color response, due to the presence of a large number of monoacylated and diacylated forms of cyanide and paeoniflorin in their structure [8]. Therefore, it is an appealing possibility to assemble PPA into the film matrix to fabricate smart films with the functionality of seafood freshness monitoring, but they cannot meet the requirements of smart films for antibacterial properties. To address this disadvantage, an attractive approach is to introduce antimicrobial agents to the film. Antimicrobial agents combat the microorganisms responsible for contamination and product deterioration [9]. Films incorporated with antimicrobial agents have been used to extend the shelf-life of food. For instance, Yong et al. [10] designed an antibacterial/antioxidant biocomposite film using soy protein isolate/sodium alginate/ε-polylysine/tannic acid. The shelf life of beef covered with the SS-ε-PL-TA film was extended by 3 days by decreasing lipid oxidation and inhibiting bacterial growth. In addition, Ying et al. [11] prepared gelatin-based antibacterial films with OEO/β-CDs for use in the chilling preservation of grass carp fillets. Due to the excellent antimicrobial properties of oregano essential oil, the films effectively prevented the increase in the TVB-N and TBA value of the refrigerated fillets and significantly inhibited the growth of spoilage organisms, thus extending the shelf life by 2–3 days. The essential oil extracted from *Amomum tsao-ko* (AEO) is also of interest. *Amomum tsao-ko* is a well-known plant-derived spice that has both medicinal and edible value and is widely distributed in China, Vietnam, and Laos [12]. Many previous studies have shown that *A. tsao-ko* has multiple pharmacological effects, such as antibacterial properties and immunity enhancement, and its active substance is mainly a volatile essential oil [13,14]. From the technological and economic points of view, AEO can be explored as a potent natural antimicrobial preservative ingredient for the food industry. Most studies on AEO have focused on its pharmaceutical properties, but there is no published research on the use of AEO for the development of smart films.

Herein, a multi-functional smart film was prepared, based on a polyvinyl alcohol/cellulose nanocrystal (PVA/CNC) matrix embedded with AEO and PPA. PVA was used to assemble the film matrix due to its film-forming properties and biodegradability. The inclusion of CNC enhanced the performance of the PVA film, such as a UV-vis barrier, mechanical properties, and moisture resistance, which has been verified in our previous research [15]. To our knowledge, this study is the first to investigate the combination of these active components in a PVA/CNC matrix, particularly the use of AEO as a novel material for developing colorimetric pH-sensitive and active food packaging. However, the performance of packaging films prepared by incorporating AEO and PPA remains unclear. Meanwhile, it remains uncertain whether PPA retains its pH-mediated color response properties in the presence of AEO. The structural, physical, and mechanical properties, antioxidant and antimicrobial activities, and pH/NH_3_-responsive performance of the obtained packaging films were analyzed, and the potential application of the target film (PVA/CNC-AEO-PPA) in mantis shrimp preservation and freshness monitoring was investigated.

## 2. Materials and Methods

### 2.1. Materials

CNC was procured from ScienceK Co., Ltd. (Huzhou, China). PVA, calcium chloride, and glycerol were purchased from Macklin (Shanghai, China). *Amomum tsao-ko* fruits were harvested from Maji Township (Fugong County, Nujiang Prefecture, Yunnan Province, China). Live mantis shrimp and purple potatoes were purchased from a local market (Zhanjiang, China). *Shewanella putrefaciens* ATCC BAA-1097 (*S. putrefaciens*) was acquired from the Guangdong Microbial Strain Conservation Center, while *Vibrio parahaemolyticus* ATCC 33,847 (*V. parahaemolyticus*) was obtained from the Guangdong Ocean University College of Food Science and Technology. DPPH, ABTS, ethanol, methanol, hydrochloric acid, ascorbic acid, and anhydrous sodium sulfate were purchased from Aladdin Biochemical Technology Co., Ltd. (Shanghai, China). Luria–Bertani (LB) broth medium and LB agar medium were purchased from Huankai Microbial Sci. & Tech. Co., Ltd. (Guangzhou, China).

### 2.2. AEO Extraction and GC-MS Analysis

Dried *Amomum tsao-ko* fruits were milled and distilled for 2 h with a Clevenger-type apparatus. The acquired AEO was dehydrated using anhydrous sodium sulfate and then stored away from light.

The volatile constituents of AEO were analyzed with a GCMS-QP2010 Plus instrument equipped with a quadrupole mass analyzer (Shimadzu, Kyoto, Japan) using an HP-5 MS column (30 m × 0.25 mm × 0.25 μm, Agilent, Santa Clara, CA, USA). The GC injection and MS interface temperatures were both maintained at 250 °C. Electron ionization (EI) was used as the ion source, the electron impact energy was 70 eV, and the ion source temperature was 230 °C. The following temperature program was used with 1 min of solvent delay. Initially, the temperature began at 60 °C (held for 1 min) and then increased to 150 °C at a rate of 6 °C/min, and then gradually increased to 240 °C (held for 5 min) at a rate of 8 °C/min using a non-split injection mode. The constant flow rate of the carrier gas (helium) was 1 mL/min. The EI mass spectra were set to scan from 45 to 550 atomic mass units (*m*/*z*).

### 2.3. Extraction and Characterization of PPA

PPA was extracted by immersing 50 g of dried purple potato powder into 500 mL ethanol-HCl solution (80%, *v*/*v*) and stirred at 600 rpm under 60 °C for 2 h; the filtrate was collected with a vacuum filtration device, then rotary-evaporated at 40 °C and dried using a freeze dryer to obtain the PPA. The total flavonoid content (TFC), total phenolic content (TPC), and total anthocyanin content (TAC) of the PPA were measured by aluminum chloride colorimetric, Folin–Ciocalteu, and pH-differential assays, respectively [16].

### 2.4. pH-Sensitivity of PPA

The pH-sensitivity assay was carried out by dissolving 10 mg of PPA into 14 mL of buffer solution (pH 2–13) and the UV-Vis spectra were collected using a spectrophotometer from 400 to 800 nm [17].

### 2.5. Fabrication of Smart Films

First, 4.5 g of PVA and 0.5 g of CNC were dissolved in 100 mL of 90 °C deionized water, with vigorous stirring at 1500 rpm for 30 min. Afterward, 0.4 g of AEO, 0.6 g of PPA, and both of them were individually added into PVA/CNC solutions, then stirred at 500 rpm for 2 h to obtain the PVA/CNC-AEO, PVA/CNC-PPA, and PVA/CNC-AEO-PPA film solutions. Then, 1 g of glycerol was incorporated as a plasticizer and homogenized using an Ultra-Turrax (IKA Works GmbH & Co, Staufen, Germany) at 12,000 rpm for 2 min (Table 1). Finally, the film-forming suspensions were evenly cast onto PTFE plates and dried at 37 °C for 8 h. The prepared films were detached from the casting plate and conditioned at 25 °C and 53% RH before further analysis.

### 2.6. Characterization of Smart Films

#### 2.6.1. Structure Characterization

The interactions among molecules of smart films matrix were obtained by FT-IR (Thermo Nicolet iN10, Thermo Fisher Scientific, Waltham, MA, USA) within the range of 500 to 4000 cm^−1^. The crystal structure of the smart films was tested using an X-ray diffractometer (D8 ADVANCE, Bruker, Karlsruhe, Germany) with CuKα radiation in the range of 2θ = 5°–60°. The thermal behavior was recorded with a thermogravimetric analyzer (DSC-PT1000, LINSEIS, Selb, Germany). Each film was weighed at approximately 10 mg, put in a sealed aluminum pan, and heated from 30 °C to 600 °C under N_2_ (20 mL/min); the heating rate was 10 °C/min [18].

#### 2.6.2. Light Transmittance and Color Evaluation

The light transmittance of the smart films was tested by UV-vis spectrophotometry at 300–800 nm. The opacity was gained according to Equation (1):

(1) $$\mathrm{Opacity}=A_{600}/L\;$$

where A_600_ represents the absorbance at 600 nm, and L represents the film thickness (mm).

Then, a colorimetric analysis of the smart films was carried out using a Color i5D (X-Rite Inc., Grand Rapids, MI, USA).

#### 2.6.3. Thickness and Barrier Properties

The thickness of the smart films was determined using a digital micrometer. The water vapor permeability (WVP) of the films was assessed, based on the method used previously [15]. The oxygen permeability of the smart films was tested according to a previous study [19]. Briefly, smart films were attached to a 50 mL conical flask containing fresh corn oil (30 mL). Afterward, the conical flask was kept at 60° C for 10 days. The oxygen permeability of the film was determined indirectly by calculating the peroxide value (PV) of the corn oil, which was measured by the sodium thiosulfate titration method.

#### 2.6.4. Mechanical Properties

Tensile tests were carried out using a tensile testing machine (Yibo Instrument Co., Ltd., Guangzhou, China). Film samples (8 × 1 cm^2^) were clamped and stretched at 6 cm/min until film breakage. Tensile strength (TS) and elongation at break (EAB) were calculated by the following equation:

(2) $$TS\;(MPa)=\frac{P}{b\times d}$$

(3) $$E\;(\%)=\frac{L-L_{0}}{L_{0}}\times 100\%$$

where P is the maximum force (N), b is the thickness (mm), d is the width of the film (mm); L is the length of the film when it breaks (mm), and L_0_ is the initial length of the film (mm).

#### 2.6.5. Antibacterial Properties

The antibacterial activity of the smart films against *S. putrefaciens* and *V. parahaemolyticus* were evaluated by the inhibition rate method [20]. Each UV-sterilized film piece of 0.1 g in weight was immersed thoroughly in 5 mL of bacterial solution (10^6^ CFU/mL) and shaken at 37 °C for 6 h. Afterward, the 0.1 mL serial dilution was spread uniformly on an LB agar plate. The plates were placed in incubators at 37 °C for 24–48 h. Additionally, bacterial morphologies treated with smart films were observed using SEM to investigate their potential antibacterial mechanism. Sterile water was used as the control group. The inhibition rate was computed according to Equation (4):

(4) Inhibitionrate(%)=((A−B)/A)×100%

where A is the colonies of the blank group and B is the colonies of a group treated with smart films.

#### 2.6.6. Antioxidant Capacity

The antioxidant activity of the films was estimated using the DPPH and ABTS radical scavenging method [21,22].

Different film amounts (0, 4, 8, 12, 16, and 20 mg) were immersed in 4 mL of DPPH methanol solution (100 µM) and stored in the dark at 25 °C for 1 h; the supernatant was collected and tested at 517 nm. Ascorbic acid and methanol were used as positive and negative controls, respectively. The equation can be expressed as follows:

(5) $$\mathrm{DPPH}\;\mathrm{scavenging}\;\mathrm{ability}\;\left(\%\right)=(1-\frac{A_{1}-A_{2}}{A_{0}})\;\times 100$$

where A_1_ is the absorbance of the DPPH solution treated with film samples, and A_2_ and A_0_ represent the absorbance of the positive and negative control groups, respectively.

Different film amounts (0, 4, 8, 12, 16, and 20 mg) were dissolved in 0.6 mL of deionized water; afterward, 3.4 mL of ABTS solution (OD_734 nm_ = 0.70) was added to 0.6 mL of sample solution, with the dark reaction continuing for 6 min. The absorbance was tested at 734 nm. Ascorbic acid and deionized water were used as positive and negative controls, respectively. The equation can be expressed as follows:

(6) $$\mathrm{ABTS}\;\mathrm{scavenging}\;\mathrm{ability}\;\left(\%\right)=(1-\frac{A_{1}-A_{2}}{A_{0}})\;\times 100$$

where A_1_ is the absorbance of the ABTS solution treated with film samples, and A_2_ and A_0_ represent the absorbance of the positive and negative control groups, respectively.

#### 2.6.7. pH/NH_3_ Responsiveness of Smart Films

The pH/NH_3_-sensitive properties of the PVA/CNC-AEO-PPA film were evaluated according to our previous study [15].

### 2.7. Application of Smart Film for Mantis Shrimp Preservation and Freshness Monitoring

The application of the target film (PVA/CNC-AEO-PPA) in mantis shrimp preservation and freshness monitoring was validated. The films (3 cm × 3 cm) were affixed on the upper surface of the inside of sterile Petri dishes (diameter: 90 mm) containing fresh mantis shrimp (30 g), sealed with parafilm, and then stored at 4 °C for 8 days. The color change of the films was observed at 2-day intervals. The freshness of the mantis shrimp was determined by testing the TVB-N and pH [23]. The TVB-N of the mantis shrimp was tested according to GB 5009.228-2016 (semi-micro method) [24].

### 2.8. Statistical Analysis

All samples were measured at least in triplicate, and the results are expressed as the mean ± standard deviation (SD). SPSS 20.0 software and a one-way analysis of variance (ANOVA) were employed for statistical analysis. Duncan’s multiple range test at a 5% significance level was used for comparison.

## 3. Results and Discussion

### 3.1. Chemical Composition of the AEO

The AEO extracted was pale yellow in color, with a yield of 1.8 ± 0.1% (*v*/*w*) based on dry weight. A total of 23 compounds (relative peak area > 0.5%) were identified by GC-MS, corresponding to 94.54% of the total mass (Table 2 and Figure S1 in the Supplementary Materials). AEO was composed of 8 monoterpenoids (15.95%), 8 oxygenated monoterpenoids (44.42%), 1 oxygenated sesquiterpene (2.53%), 5 aliphatics (20.78%) and 1 aromatic (10.86%). The major compounds were determined as 1,8-cineole (19.03%), 4-propylbenzaldehyde (10.86%), tran-2-Decenal (10.00%), geranial (9.96%), and citral (7.55%). Similar results were reported by Liu et al. [14], and their quantitative and qualitative differences from the current study could be attributed to the geographical origin of the plant and its genetic and other factors.

### 3.2. Characterization of PPA

**Figure 1:**
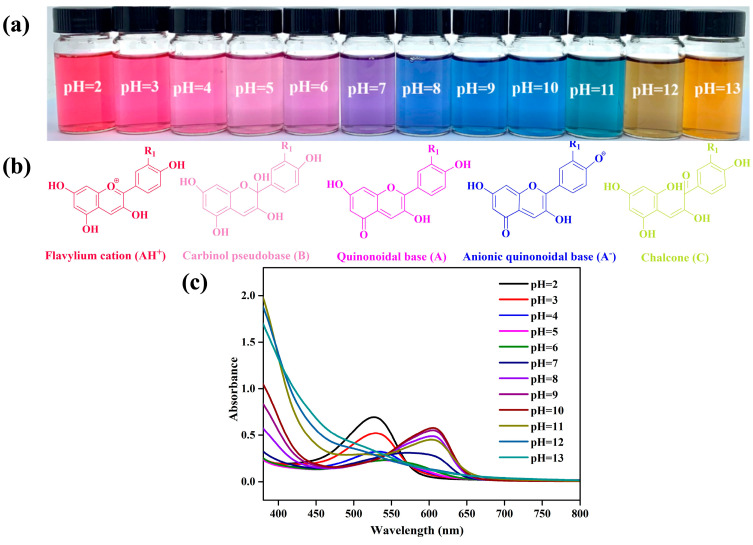
Photographs (**a**), structural transformation (**b**), and UV-vis spectra (**c**) of PPA in pH buffer solutions (2–13).

The color responses of the PPA to the pH solutions and their corresponding UV-vis spectra are depicted in Figure 1. The PPA displayed distinct colors in various pH buffer solutions (Figure 1a). The changes in color and the corresponding peaks were attributed to conformation variations of the anthocyanin structure. At a pH below 3, the bright red color of PPA resulted from the structural form of the flavylium cation (AH^+^). Light pink was observed within a pH range of 4–6. The nucleophilic attack of the water molecules on the 2-position carbon atom of the anthocyanin B ring caused the breaking of the double bond; as a result, the conjugate system was destroyed and a colorless carbinol pseudobase (B) was formed, and the absorbance gradually decreased. By increasing the pH to 7.0, the structure of the PPA changed to a quinonoidal base (A), resulting in a color shift to purple. As the pH increased to 8, the color of the PPA solution turned blue, which was attributed to the PPA structure changing to a blue anionic quinonoidal base (A^−^) [7]. The formation of chalcone (C) led to a color shift toward yellow at pH 12. With the pH changed from acidic to alkaline, the maximum absorption peak of PPA shifted from 527 nm to 605 nm. From the above results, it can be seen that the PPA can respond to pH changes and holds great promise for replacing synthetic dyes in smart films to monitor food quality.

### 3.3. Characterization of Smart Films

#### 3.3.1. FT-IR

**Figure 2:**
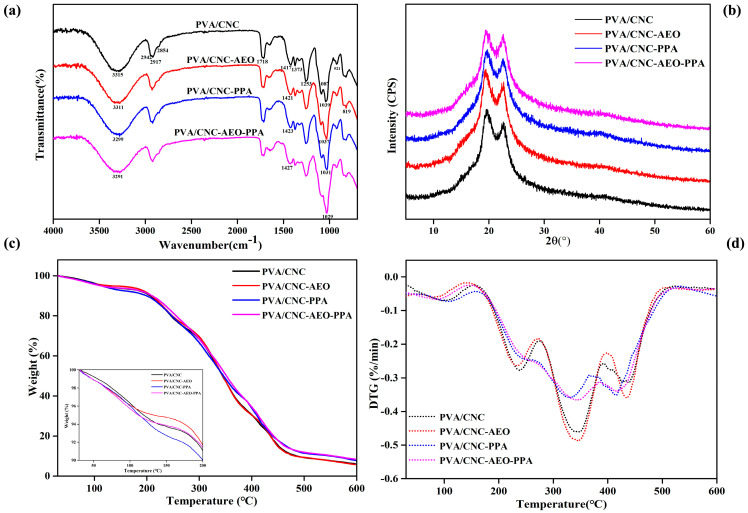
FTIR spectra (**a**), XRD patterns (**b**), and TGA (**c**) and DTG (**d**) profiles of smart films.

From the FTIR data (Figure 2a), no new bands were observed in the films after the addition of PPA or/and AEO, suggesting that PPA and AEO had no effect on the chemical structure of the PVA/CNC film. For the PVA/CNC film, the bands at 3315 cm^−1^ and 1039 cm^−1^ might be attributed to O-H and C-O stretching, respectively [25]. The bands at 2942, 2917, and 2854 cm^−1^ can be assigned to the symmetric and asymmetric stretching vibrations of CH from methyl or methylene groups.

**Figure 3:**
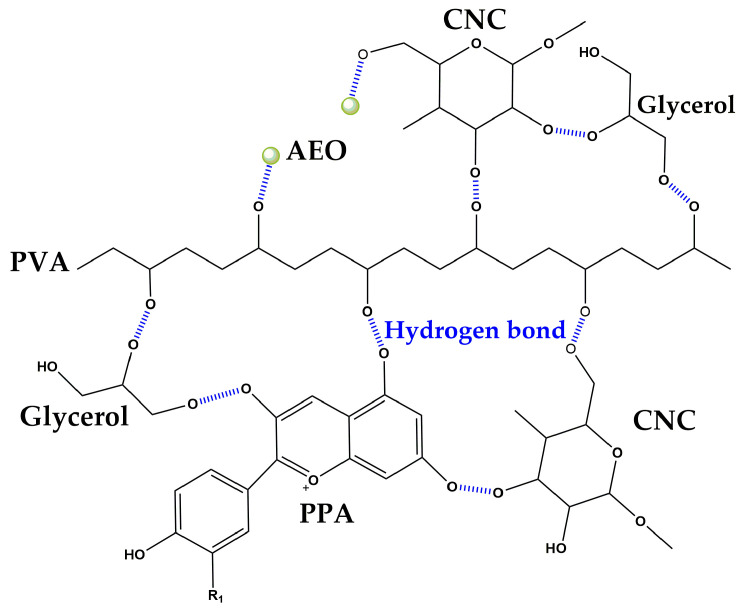
Possible interactions between the functional groups of each compound.

With the addition of PPA or/and AEO, the -OH stretching of the PVA/CNC at 3315 cm^−1^ shifted lower to 3311 cm^−1^ (PVA/CNC-AEO), 3299 cm^−1^ (PVA/CNC-PPA), and 3291 cm^−1^ (PVA/CNC-AEO-PPA), respectively. Meanwhile, lower shifts were observed on C-O stretching to 1037 cm^−1^ (PVA/CNC-AEO), 1031 cm^−1^ (PVA/CNC-PPA), and 1029 cm^−1^ (PVA/CNC-AEO-PPA), respectively, suggesting the formation of intermolecular hydrogen bonds between the hydroxyl groups of AEO, PPA, and PVA/CNC, as vividly described in Figure 3 [26]. The intensity of the absorption band intensity at 3315 cm^−1^, 1718 cm^−1^, 1253 cm^−1^, and 1087 cm^−1^ reduced, which might be related to the interactions among PPA, AEO, and the hydroxyl groups of PVA/CNC [27]. The peaks at 1031 cm^−1^ and 1029 cm^−1^ turned out to be sharper and more visible in the PVA/CNC-PPA and PVA/CNC-AEO-PPA films; the aromatic ring deformation of the PPA was responsible for this change, verifying the presence of PPA in the smart film and the formation of strong interactions.

#### 3.3.2. XRD

Figure 2b shows the effect of PPA and AEO addition on the crystallinity of smart films. It can be seen that all films show typical diffraction patterns with strong signals at 19.5° and 22.5°, corresponding to the crystal structures of PVA and CNC, respectively. Meanwhile, no characteristic peak was formed in the XRD diffraction spectrum, suggesting that the addition of PPA and AEO did not change the crystal structure of the PVA/CNC films. Nevertheless, the diffraction peak intensity at 22.5° increased with the addition of PPA or/and AEO. The crystallinity indexes of PVA/CNC, PVA/CNC-AEO, PVA/CNC-PPA, and PVA/CNC-AEO-PPA were 37.16%, 39.29%, 38.06%, and 39.73%, respectively. This might be explained by the formation of hydrogen bonds between PVA/CNC and PPA or AEO through intermolecular interactions, which is consistent with the results reported in previous literature [28].

#### 3.3.3. TGA

The thermal stability of the smart films was tested by TGA/DTG (Figure 2c,d). All films exhibited a multi-step thermal degradation mode. The first step of degradation (30–151 °C) was assigned to the evaporation of the free and bound water absorbed in the film. Moreover, some degradation of AEO and PPA might occur in this step [29]. The second stage of thermal decomposition appeared around 160–273 °C, which was due to the thermal degradation of glycerol [30]. The most significant weight loss, observed in the third stage (276–394 °C), was associated with the degradation of PVA and CNC. The maximum decomposition temperature (Tmax) for smart films was at approximately 344 °C (PVA/CNC film), 344 °C (PVA/CNC AEO film), 329 °C (PVA/CNC-PPA film), and 345 °C (PVA/CNC-AEO-PPA film). The final residual weight of the PVA/CNC, PVA/CNC-AEO, PVA/CNC-PPA, and PVA/CNC-AEO-PPA films at 600 °C was 6.16%, 5.78%, 7.78%, and 8.44%, respectively. The TGA profiles indicate that the thermal stability of the PVA/CNC-AEO-PPA film was better than that of the PVA/CNC-AEO and PVA/CNC-PPA films. Surprisingly, the loss for all films below 200 °C was about 10%, confirming their good thermal stability and ability to meet the requirements of food packaging.

#### 3.3.4. Colorimetric and Optical Properties

**Figure 4:**
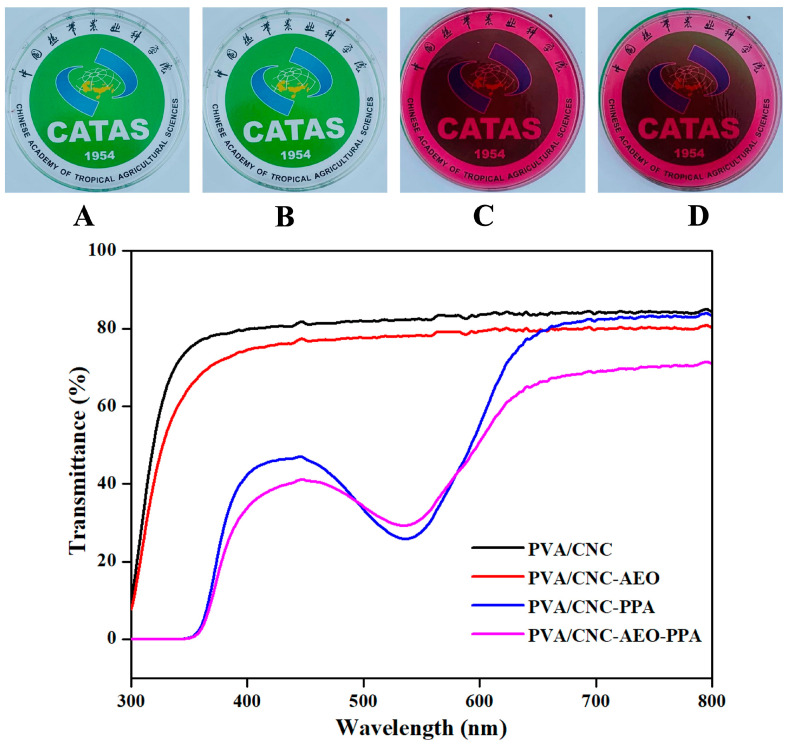
Photograph of PVA/CNC (**A**), PVA/CNC-AEO (**B**), PVA/CNC-PPA (**C**), and PVA/CNC-AEO-PPA (**D**) film, and the UV-vis light transmittance of smart films.

As shown in Figure 4, high transparency could be seen in the PVA/CNC and PVA/CNC-EO films. Interestingly, the transmittance of all films decreased dramatically after the addition of PPA, exhibiting a strong blockage of UV radiation. There was a significant decrease in light transmission at 300–400 nm, which was caused by the absorption of UV light by the PPA [31]. The phenolic compounds showed good UV absorption potential due to their rich aromatic rings [32]. In addition, the transmittance curve was bent at 500–600 nm in PPA films, which might be ascribed to the partial absorption of light by the PPA. The abundant chromophiles, such as C-C and C-O, in anthocyanins contributed greatly to the decreased light transmittance of PPA films. Similar conclusions were obtained by other studies [33,34]. Correspondingly, the PVA/CNC film exhibited the lowest opacity. Higher opacity was seen in the PPA film, which was consistent with the results in Table 3.

The color parameters of the films are shown in Table 3. It can be seen that the L* parameter of the PVA/CNC film was nearly 90, so it tended toward lightness. The addition of AEO showed no significant effect on the appearance of the film. The L*-value of the PVA/CNC-PPA film decreased significantly to 44.62 ± 0.08 (*p* < 0.05), while the a*-value significantly increased from −0.68 ± 0.15 to 48.24 ± 0.13, suggesting that the film gradually turned red (*p* < 0.05). The ΔE also increased significantly (*p* < 0.05), which was in accordance with the films’ appearance (Figure 4).

#### 3.3.5. Thickness

**Figure 5:**
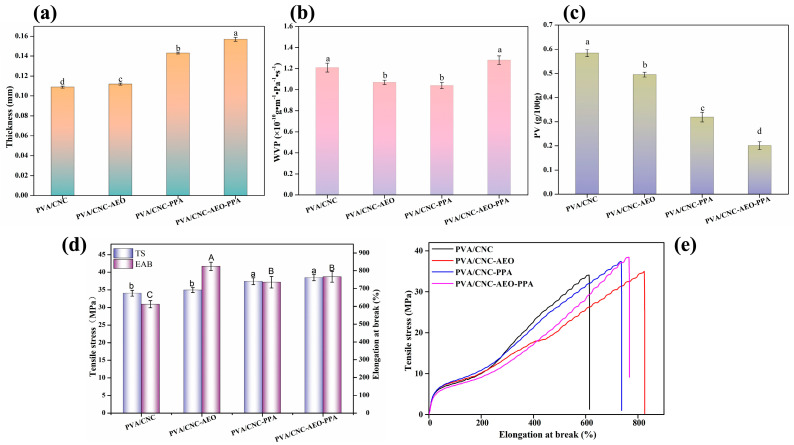
Graphs showing the thickness (**a**), WVP (**b**), PV values (**c**), TS and EAB (**d**), and the tensile stress-strain curves (**e**) of the smart films. Different letters between groups indicated a significant difference (*p* < 0.05).

The thickness of the smart films ranged from 0.109 mm to 0.157 mm (Figure 5a). Compared with the PVA/CNC film, the thickness of the PVA/CNC-AEO films increased significantly (*p* < 0.05). This might be because oil droplets occupy a certain volume, increasing the free volume of the film network, the mobility of macromolecules, and the thickness of the film [21]. Similarly, the inclusion of PPA resulted in an increase in the thickness of the PVA/CNC film (*p* < 0.05), which can be ascribed to the rise in the dry solids content of the smart films [35].

#### 3.3.6. WVP

As illustrated in Figure 5b, the WVP of the PVA/CNC film reached (1.21 ± 0.05) × 10^−10^ g·m^−1^·Pa^−1^·s^−1^. The addition of the hydrophobic component of AEO improved the water barrier properties of the PVA/CNC-AEO film. Studies have reported that some substances with emulsifying properties increase the dispersion of hydrophobic essential oils, thus increasing the curvature of the diffusion channels and the steric hindrance effect of the water molecules. These factors improve the water-proofing properties of the films [19]. When the PPA was blended, the rich hydroxyl groups in the PPA could form intermolecular hydrogen bonds with the hydrophilic hydroxyl groups in PVA/CNC, thereby reducing the interactions between hydrophilic hydroxyl groups in the PVA/CNC and water molecules [17,36]. Nevertheless, the WVP of the PVA/CNC-AEO-PPA film reached (1.28 ± 0.04) × 10^−10^ g·m^−1^·Pa^−1^·s^−1^. This may be due to the fact that the addition of AEO and PPA simultaneously disrupted the compact structure of the smart film, leading to an increase in the water vapor transmission rate of the smart film [37]. Ultimately, there was only a limited difference in WVP between the PVA/CNC-AEO-PPA and PVA/CNC films (*p* > 0.05). The WVP of the PVA/CNC-AEO-PPA film was similar to that reported by Wu et al. [38] and was higher than that reported by Yao et al. [39]. The difference may be due to the addition of different types of polysaccharides or plant extracts.

#### 3.3.7. Oxygen Permeability (OP)

The restriction of oxygen transfer works by delaying the oxidation rate and deterioration of food products [40]. The OP of the smart films was reflected indirectly by the PV of the oil. The PVA/CNC films exhibited the highest PV (Figure 5c). Following the addition of PPA, the PV decreased from 0.584 ± 0.04 g/100 g to 0.319 ± 0.02 g/100 g. Meanwhile, the incorporation of the AEO reduced the PV to 0.495 ± 0.02 g/100 g. These results indicate that the oxygen-barrier capacities of the film were effectively enhanced, which could be owing to certain factors. Firstly, the hydrogen bond interactions between the PPA and PVA/CNC matrix and the well-dispersed PPA and AEO restrained or prolonged the oxygen penetration path. Secondly, the PPA possessed excellent oxygen scavenging ability and could trap some oxygen molecules in the matrix, thereby reducing the OP [21]. As expected, the synergistic effect of the AEO and PPA resulted in the best oxygen barrier properties of the PVA/CNC-EO-PPA film.

#### 3.3.8. Mechanical Properties

The stress-strain curves of smart films are illustrated in Figure 5d,e. The TS and EAB of the PVA/CNC film were 34.05 MPa and 612.18%, respectively, suggesting that the PVA/CNC film possessed good flexibility (high EAB) and strong strength (high TS). When AEO was blended, there was only a limited difference (*p* > 0.05) in TS between the PVA/CNC-AEO film and PVA/CNC film, while the EAB of the PVA/CNC-AEO film increased significantly. This occurrence could be explained by the fact that the plasticizing effect of the AEO promoted the sliding of the chain, contributing greater flexibility and fluidity to the chain [41]. The TS and EAB of the PVA/CNC film were significantly (*p* < 0.05) enhanced by PPA addition. The improved mechanical properties could be ascribed to the homogeneous dispersion of the PPA in the film matrix and the intermolecular interactions between the anthocyanins, PVA, and CNC. The TS and EAB of the PVA/CNC-AEO-PPA film were 38.45 MPa and 765.85%, respectively, indicating that the mechanical properties of the PVA/CNC film could be enhanced effectively by the addition of PPA and AEO.

#### 3.3.9. Antibacterial Activity

**Figure 6:**
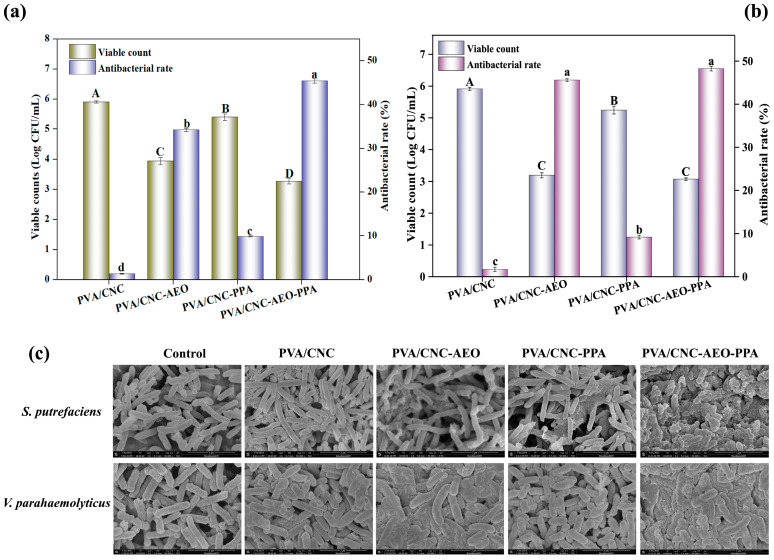
Antibacterial activity against *S. putrefaciens* (**a**) and *V. parahaemolyticus* (**b**) of the smart films; (**c**) SEM images of *S. putrefaciens* and *V. parahaemolyticus* when treated with smart films. Different letters between groups indicated a significant difference (*p* < 0.05).

*V. parahaemolyticus* and *S. putrefaciens* are common and predominant bacteria in seafood, which is prone to food spoilage and the corrosion of food processing equipment, causing severe health problems and enormous economic losses [42]. The antibacterial activity of smart films against *S. putrefaciens* and *V. parahaemolyticus* was evaluated and the antibacterial rate is shown in Figure 6a,b. When PPA was incorporated into the PVA/CNC film, the viability of *S.putrefaciens* decreased from 5.99 ± 0.08 log CFU/mL to 5.40 ± 0.07 log CFU/mL, and the viability of *V. parahaemolyticus* reduced from 6.05 ± 0.03 log CFU/mL to 5.24 ± 0.22 log CFU/mL. The antibacterial rate of the PVA/CNC-PPA film against *S. putrefaciens* and *V. parahaemolyticus* reached 9.85% and 9.15%, respectively. This may be because the active polyphenols could enter the bacterial cell membranes and bind to the cellular proteins, thereby inactivating the proteins and limiting bacterial growth [43]. Of note, PVA/CNC-AEO exhibited better antibacterial activity compared with the PVA/CNC-PPA film. When treated with the PVA/CNC-AEO film, the viability of *S. putrefaciens* decreased to 3.94 ± 0.07 log CFU/mL, and the viability of *V. parahaemolyticus* reduced to 3.19 ± 0.08 log CFU/mL. The antibacterial rates of PVA/CNC-AEO against *S. putrefaciens* and *V. parahaemolyticus* were 34.22% and 45.59%, respectively. The superior antimicrobial activity was ascribed to AEO as the main antibacterial component, which could alter the permeability and integrity of cell membranes, resulting in the leakage of nucleic acids and proteins [13]. As expected, PVA/CNC-AEO-PPA showed the optimal antibacterial effect. The bactericidal rates of PVA/CNC-AEO-PPA against *S. putrefaciens* and *V. parahaemolyticus* reached 45.41% and 48.25%, respectively. The SEM images showed that the bacterial structures underwent collapse, contraction, and adhesion after treatment with PVA/CNC-AEO and PVA/CNC-AEO-PPA, leading to the death of the bacteria (Figure 5c). However, the morphology of the bacteria treated with PVA/CNC-PPA hardly changed, which was consistent with its limited antibacterial activity. The above results proved the potential of PVA/CNC-AEO-PPA films to extend the shelf life of food products.

#### 3.3.10. Antioxidant Activity

The TAC of the PPA dry extract was 9.69 mg/g. The TPC and TFC of the PPA were 77.21 mg GAE/g and 179.37 mg QE/g, respectively. Phenolic compounds are the most plentiful secondary metabolites in plants, with potent hydrogen-donating or free-radical scavenging capability, suggesting the great potential of PPA as an antioxidant [44].

Packaging materials with free radical scavenging attributes can protect food from spoilage and oxidative damage [45]. The antioxidant activity of smart films was evaluated based on the DPPH and ABTS methods. Different methods of antioxidant activity were utilized since they complement each other. The DPPH assay was used to detect the antioxidant capacity of samples following the single electron transfer (SET) and hydrogen atom transfer (HAT) mechanisms, while the ABTS assay was used to evaluate the antioxidant capacity of the samples following the HAT mechanism.

**Figure 7:**
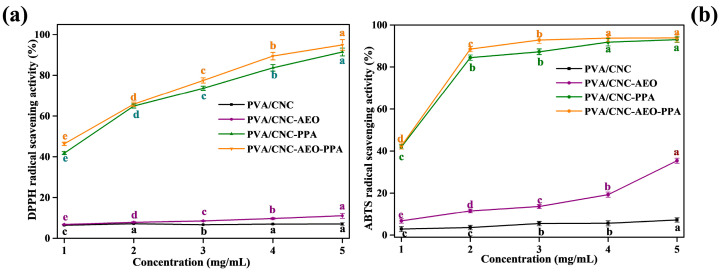
DPPH (**a**) and ABTS (**b**) radical scavenging activity of smart films. Different letters between groups indicated a significant difference (*p* < 0.05).

As seen in Figure 7, the PVA/CNC film revealed poor antioxidant activity due to the lack of effective antioxidant components, while the scavenging capability of PVA/CNC films on the DPPH and ABTS radical were significantly enhanced by PPA addition (*p* < 0.05). Potent antioxidant activity was related to PPA, which comprised positively charged compounds (flavylium cations) at the oxygen atom of the C-ring of the flavonoid skeleton that would promote the transfer of hydrogen atoms for radical inhibition [46]. A significantly higher DPPH radical scavenging ability of 91.43% and relatively higher ABTS radical scavenging ability of 92.95% were found in 5 mg/mL of the PVA/CNC-PPA film. The above results are probably due to the fact that both PPA and AEO possess phenolic compounds that can contribute hydrogen atoms. Simultaneously, the obtained radical scavenging ability in the ABTS assay was higher than that in the DPPH assay. The reason might be that DPPH radicals are more sensitive to the reaction environment; small changes in solvent, pH, or temperature might have a more significant effect on the measurement results, leading to higher variability. In addition, the absorbance of the DPPH radical decreased in the presence of light, and the ABTS assay was less susceptible to the interference of PPA color due to the higher wavelength used [46].

#### 3.3.11. pH-Sensitivity Properties

The color response of the PVA/CNC-AEO-PPA film was distinguishable at pH 2–13 (Table 4), with a red color at pH 2, a light pink at 3–5, a purple color at pH 6–7, blue at pH 8–11, and a green-yellowish color at pH 12–13, which was consistent with that of the PPA solutions (Figure 1). It was observed that the a* value decreased gradually as the pH increased from 2 to 11, suggesting a decrease in the red color, while the b* value altered from positive to negative, indicating that the film turned blue. With the pH changing from 11 to 13, an increasing b* value was observed, resulting in a yellow color. The ΔE of the PVA/CNC-AEO-PPA film gradually increased with increasing pH. Usually, the pH of aquatic products in the process of corruption is between 7.0 and 8.0 [47]. Impressively, the PVA/CNC-AEO-PPA film presented a relatively large color variation at pH 7–8 with ΔE > 5 (easily distinguished by the naked eye) [48]. Therefore, the PVA/CNC-AEO-PPA film is expected to work as a pH colorimetric indicator for monitoring seafood freshness.

#### 3.3.12. Volatile Ammonia Response

The color response to volatile ammonia is a key characteristic of an indicator film before it is applied in seafood freshness testing. Therefore, the colorimetric change of the PVA/CNC-AEO-PPA film in response to ammonia was measured to simulate the release of volatile gas during the seafood deterioration process (Table 5). After exposing the PVA/CNC-AEO-PPA film to ammonia water (10 mM), significant color changes (red→blue) occurred rapidly within 30 min (ΔE > 20), accompanied by variations in the a* value (45.22→7.31) and b* value (−2.08→−6.57). The underlying mechanism of the visual color variation of the smart films was due to the structural change of PPA upon contact with ammonia molecules. OH^−^ was formed when the ammonia molecules combined with the H_2_O contained in the film [4], further triggering the color change in the PVA/CNC-AEO-PPA film. These results proved the color response of the smart film to the volatile ammonia compounds produced in seafood.

### 3.4. Application of Smart Film for Mantis Shrimp Preservation and Freshness Monitoring

The PVA/CNC-AEO-PPA film was applied for mantis shrimp preservation and freshness monitoring owing to its superior comprehensive performance, including its mechanical strength, barrier properties, antibacterial activity, antioxidant capacity, and pH/NH_3_ sensitivity. According to previous reports, the freshness of raw shrimp can be classified into four categories, based on TVB-N: fresh (<12 mg/100 g), edible but slightly decomposed (12–20 mg/100 g), borderline (20–25 mg/100 g), and spoiled (>25 mg/100 g) [3].

**Figure 8:**
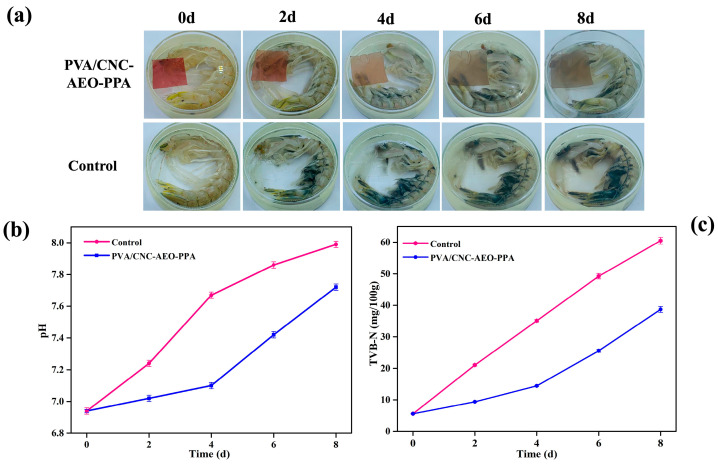
PVA/CNC-AEO-PPA film use for mantis shrimp freshness monitoring (**a**); the corresponding levels of pH (**b**) and TVB-N (**c**).

In the control group, the initial TVB-N for mantis shrimp was 5.59 mg/100 g, followed by an increase to 21.04 mg/100 g at 2 days, and 35.09 mg/100 g at 4 days. The shrimp shells visibly began to blacken at 2 days, indicating that the mantis shrimp was not suitable for consumption after 2 days (Figure 8). A series of color changes occurred on the PVA/CNC-AEO-PPA film: rose red (0 days)—light red (2 days)—pink (4 days)—light gray (6 days)—dark gray (8 days). Initially, the TVB-N was 5.59 mg/100 g (rose red), increased to 25.57 mg/100 g (light gray) at 6 d, and to 38.69 mg/100 g (dark gray) at 8 days, implying that the mantis shrimp was completely spoiled at 6 d due to the greatly elevated ΔE values (a value over 26; Table 6). Compared with the control group, PVA/CNC-AEO-PPA film could prolong the quality of mantis shrimp for 4 days at 4 °C, indicating its effective preservation effect. After 8 days of storage, the pH of the control group gradually increased, and it was significantly higher than those of the PVA/CNC-AEO-PPA group (*p* < 0.05), which could be ascribed to the excellent antimicrobial properties of the PVA/CNC-AEO-PPA film. As a result, the PVA/CNC-AEO-PPA film could simultaneously achieve the functions of mantis shrimp preservation and freshness monitoring.

## 4. Conclusions

In this work, we introduced APP and AEO as functional agents to fabricate multifunctional smart packaging films for mantis shrimp preservation and freshness indicator applications. FT-IR and XRD analysis showed that hydrogen bonds were formed among PPA, AEO, and the film matrix. TG analysis proved the excellent thermo-stability of smart films. The incorporation of APP and AEO improved the oxygen barrier and UV-blocking properties of the PVA/CNC film. In addition, the PVA/CNC-AEO-PPA film exhibited excellent mechanical strength (TS = 38.45 MPa, EAB = 765.85%), DPPH (91.43%), and ABTS (92.95%) radical scavenging capability, as well as antimicrobial activity against *S. putrefaciens* (45.41%) and *V. parahaemolyticus* (48.25%). Importantly, the PVA/CNC-AEO-PPA film showed a high-sensitivity color response to pH/NH_3_. The trials with mantis shrimp confirmed that the PVA/CNC-AEO-PPA film could differentiate fresh and spoiled mantis shrimp according to the color change (from rose red to light gray), which could be observed with the naked eye. Meanwhile, the PVA/CNC-AEO-PPA film could effectively preserve the quality of shrimp, indicating its great potential for use as a smart film to maintain and monitor the freshness of seafood in real time.

## Figures and Tables

**Table 1:** Composition of smart films.

Sample	PVA (% *w*/*v*)	CNC (% *w*/*v*)	AEO (% *w*/*v*)	Tween-80 (% *w*/*v*)	PPA (% *w*/*v*)	Glycerol (% *w*/*v*)
PVA/CNC	4.5	0.5	-	-	-	1.0
PVA/CNC-AEO	4.5	0.5	0.4	0.1	-	1.0
PVA/CNC-PPA	4.5	0.5	-	-	0.6	1.0
PVA/CNC-AEO-PPA	4.5	0.5	0.4	0.1	0.6	1.0

**Table 2:** Volatile aroma compounds identified in AEO by GC-MS.

No.	Constituents	RT	Molecular Formula	RI		% Relative Peak Area
				RI(C)	RI(Lit)	
1	α-Pinene	5.415	C_10_H_16_	931	933	2.78
2	Sabinene	6.362	C_10_H_16_	974	974	1.56
3	β-Pinene	6.449	C_10_H_16_	977	978	1.63
4	Octanal	6.951	C_8_H_16_O	1000	1001	2.49
5	α-Phellandrene	7.040	C_10_H_16_	1005	1005	3.61
6	ρ-Cymene	7.490	C_10_H_14_	1024	1025	2.08
7	D-Limonene	7.590	C_10_H_16_	1028	1030	2.23
8	1,8-Cineole	7.759	C_10_H_18_O	1031	1032	19.03
9	β-Ocimene	8.000	C_10_H_16_	1039	1040	1.44
10	2-Octenal	8.249	C_8_H_14_O	1055	1056	2.84
11	Isoborneol	10.939	C_10_H_18_O	1161	1160	0.62
12	Terpinen-4-ol	11.208	C_10_H_18_O	1180	1180	0.82
13	α-Terpineol	11.538	C_10_H_18_O	1193	1195	2.55
14	Decanal	11.810	C_10_H_18_O	1199	1200	0.69
15	Citral	12.784	C_10_H_16_O	1213	1216	7.55
16	Geraniol	13.116	C_10_H_18_O	1246	1247	2.01
17	tran-2-Decenal	13.292	C_10_H_18_O	1260	1260	10.00
18	Geranial	13.559	C_10_H_16_O	1278	1279	9.96
19	4-Propylbenzaldehyde	14.055	C_10_H_12_O	1293	1294	10.86
20	Citral dimethyl acetal	14.505	C_12_H_22_O_2_	1320	1322	1.19
21	Geranyl acetate	16.020	C_12_H_20_O_2_	1385	1386	1.31
22	2-Dodecenal	17.849	C_12_H_2_2O	1446	1449	4.76
23	trans-Nerolidol	19.720	C_15_H_26_O	1565	1565	2.53
Total						94.54

RT: Retention times. RI(C): Calculated retention index; RI(Lit): literature retention index.

**Table 3:** Color parameters and opacity of the smart films.

Films	L*	a*	b*	ΔE	Opacity
PVA/CNC	90.60 ± 0.30 ^a^	−0.68 ± 0.15 ^c^	2.27 ± 0.24 ^b^	4.41 ± 0.27 ^c^	0.04 ± 0.01 ^d^
PVA/CNC-AEO	91.28 ± 0.21 ^a^	−0.72 ± 0.04 ^c^	2.90 ± 0.01 ^a^	4.01 ± 0.19 ^c^	0.23 ± 0.03 ^c^
PVA/CNC-PPA	44.62 ± 0.08 ^c^	48.24 ± 0.13 ^a^	−2.63 ± 0.04 ^c^	68.46 ± 0.04 ^a^	1.32 ± 0.02 ^b^
PVA/CNC-AEO-PPA	48.69 ± 0.06 ^b^	45.22 ± 0.23 ^b^	−2.08 ± 0.06 ^c^	63.40 ± 0.20 ^b^	1.67 ± 0.10 ^a^

Note: Values are given as the mean ± SD. Different letters in the same column indicate significant differences (*p* < 0.05).

**Table 4:** Color variations of the PVA/CNC-AEO-PPA film at different pH values.

Samples	pH	L*	a*	b*	ΔE	Photograph
PVA/CNC- AEO-PPA	2	45.74 ± 1.46 ^cd^	45.48 ± 0.43 ^a^	8.53 ± 0.29 ^b^	11.01 ± 0.75 ^a^	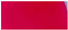
	3	41.46 ± 2.19 ^e^	35.86 ± 0.54 ^b^	3.58 ± 0.17 ^d^	13.11 ± 1.52 ^b^	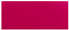
	4	40.04 ± 0.57 ^e^	34.69 ± 0.28 ^c^	3.45 ± 0.02 ^d^	14.71 ± 0.33 ^c^	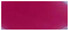
	5	48.66 ± 0.4 ^bc^	30.10 ± 0.10 ^d^	1.83 ± 0.18 ^e^	15.62 ± 0.28 ^c^	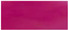
	6	55.92 ± 1.96 ^a^	20.48 ± 0.30 ^e^	2.00 ± 0.05 ^e^	26.10 ± 0.61 ^e^	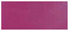
	7	48.47 ± 2.43 ^bc^	20.64 ± 0.88 ^e^	0.63 ± 0.15 ^f^	24.73 ± 0.89 ^d^	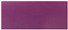
	8	51.33 ± 0.20 ^b^	11.24 ± 0.06 ^g^	−1.93 ± 0.01 ^g^	34.08 ± 0.18 ^g^	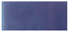
	9	50.17 ± 1.40 ^b^	11.39 ± 0.58 ^g^	−2.20 ± 0.05 ^g^	33.86 ± 0.32 ^g^	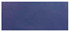
	10	45.16 ± 1.39 ^d^	13.23 ± 0.24 ^f^	−1.72 ± 0.03 ^g^	32.19 ± 0.28 ^f^	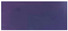
	11	39.53 ± 0.59 ^e^	6.95 ± 0.30 ^h^	−5.32 ± 0.02 ^h^	39.48 ± 0.54 ^h^	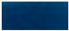
	12	29.55 ± 0.62 ^f^	2.86 ± 0.06 ^i^	4.49 ± 0.13 ^c^	46.95 ± 0.27 ^i^	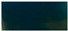
	13	50.88 ± 1.82 ^b^	20.73 ± 0.28 ^e^	38.47 ± 0.76 ^a^	47.42 ± 0.16 ^i^	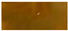

Values are given as the mean ± SD. Different letters in the same column indicate significant differences (*p* < 0.05).

**Table 5:** Color variations of PVA/CNC-AEO-PPA film treated with volatile ammonia.

Samples	Time/min	L*	a*	b*	ΔE	Photograph
PVA/CNC-AEO-PPA	0	48.69 ± 0.06 ^a^	45.22 ± 0.23 ^a^	−2.08 ± 0.06 ^a^	-	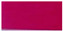
	10	44.57 ± 0.40 ^d^	33.05 ± 0.28 ^b^	−4.57 ± 0.62 ^b^	13.09 ± 0.54 ^a^	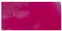
	20	46.44 ± 0.01 ^b^	20.76 ± 0.71 ^c^	−4.89 ± 0.24 ^b^	24.72 ± 0.23 ^b^	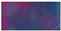
	30	45.38 ± 0.13 ^c^	7.31 ± 0.52 ^d^	−6.57 ± 0.13 ^c^	38.32 ± 0.05 ^c^	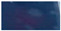

Values are given as mean ± SD. Different letters in the same column indicate significant differences (*p* < 0.05).

**Table 6:** Color parameters of the PVA/CNC-AEO-PPA film in mantis shrimp freshness monitoring.

Sample	Time (Days)	L*	a*	b*	ΔE
PVA/CNC-AEO-PPA	0	48.53 ± 0.07 ^b^	45.86 ± 0.10 ^a^	−2.10 ± 0.05 ^e^	-
	2	50.23 ± 0.06 ^a^	42.27 ± 0.04 ^b^	−1.42 ± 0.06 ^d^	4.03 ± 0.12 ^d^
	4	47.21 ± 0.10 ^c^	35.21 ± 0.05 ^c^	1.90 ± 0.03 ^c^	11.45 ± 0.31 ^c^
	6	40.28 ± 0.09 ^d^	20.67 ± 0.06 ^d^	2.89 ± 0.06 ^b^	26.97 ± 0.48 ^b^
	8	38.46 ± 0.08 ^e^	16.26 ± 0.04 ^e^	3.08 ± 0.07 ^a^	31.69 ± 0.61 ^a^

Values are given as the mean ± SD. Different letters in the same column indicate significant differences (*p* < 0.05).

## Data Availability

The original contributions presented in the study are included in the article, further inquiries can be directed to the corresponding authors.

## Supplementary Materials

The following supporting information can be downloaded at: https://www.mdpi.com/article/10.3390/foods13111638/s1, Figure S1: GC-MS chromatogram of the steam distillation of the essential oil of *Amomum tsao-ko*.
