# Supplementary figures and images for: Enhancement of Colorimetric pH-Sensitive Film Incorporating Amomum tsao-ko Essential Oil as Antibacterial for Mantis Shrimp Spoilage Tracking and Fresh-Keeping
Source: Foods. 2024 May 24;13(11):1638. doi: 10.3390/foods13111638 (PMC11171633; doi:10.3390/foods13111638)

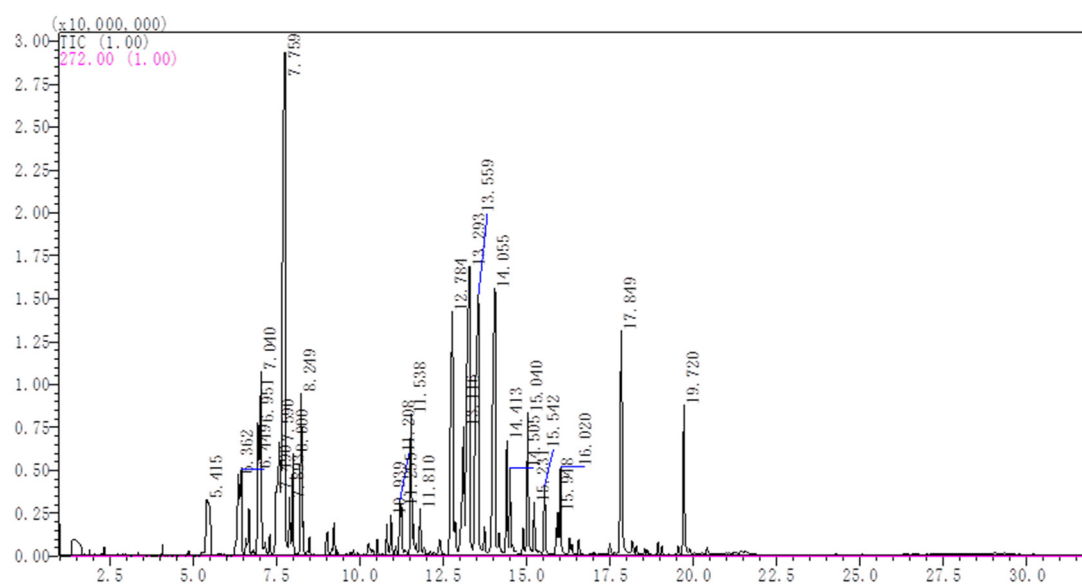

**Figure S1.** GC-MS chromatogram of steam distillation essential oil of *Amomum tsao-ko*

Supplement: Supplementary file 1 [file foods-13-01638-s001.zip › foods-3000051-supplementary.pdf]
